# Red Pepper Seeds Inhibit Hepatic Lipid Accumulation by Inducing Autophagy via AMPK Activation

**DOI:** 10.3390/nu14204247

**Published:** 2022-10-12

**Authors:** Young-Hyun Lee, Hwa-Jin Kim, Mikyoung You, Hyeon-A Kim

**Affiliations:** 1 Department of Food and Nutrition, Mokpo National University, Muan-gun 58554, Korea; 2 Nutrition Research Institute, University of North Carolina, Chapel Hill, NC 28081, USA

**Keywords:** autophagy, lipogenesis, non-alcoholic fatty liver diseases, red pepper seed, AMPK/mTOR

## Abstract

Although the red pepper and its seeds have been studied for metabolic diseases, the effects and potential mechanisms of red pepper seed extract (RPS) on hepatic lipid accumulation are not yet completely understood. This study aimed to evaluate the inhibitory effect of RPS on hepatic lipid accumulation via autophagy. C57BL/6 mice were fed a high-fat diet (HFD) or a HFD supplemented with RPS. RPS treatment inhibited hepatic lipid accumulation by suppressing lipogenesis, inducing hepatic autophagic flux, and activating AMPK in HFD-fed mice. To investigate the effect of RPS on an oleic acid (OA)-induced hepatic steatosis cell model, HepG2 cells were incubated in a high-glucose medium and OA, followed by RPS treatment. RPS treatment decreased OA-induced lipid accumulation and reduced the expression of lipogenesis-associated proteins. Autophagic flux dramatically increased in the RPS-treated group. RPS phosphorylated AMPK in a dose-dependent manner, thereby dephosphorylated mTOR. Autophagy inhibition with 3-methyladenine (3-MA) antagonized RPS-induced suppression of lipogenesis-related protein expressions. Moreover, the knockdown of endogenous AMPK also antagonized the RPS-induced regulation of lipid accumulation and autophagy. Our findings provide new insights into the beneficial effects of RPS on hepatic lipid accumulation through the AMPK-dependent autophagy-mediated downregulation of lipogenesis.

## 1. Introduction

The rapid upsurge in obesity and diabetes prevalence has contributed to a parallel increase in the worldwide prevalence of non-alcoholic fatty liver disease (NAFLD). NAFLD is currently the most common chronic liver disease characterized by the over-accumulation of lipids in the liver without significant ethanol consumption [1,2]. The NAFLD spectrum ranges from simple nonalcoholic steatosis to more severe steatohepatitis, which can progress to chronic liver diseases such as cirrhosis and liver cancer. The excessive hepatic accumulation of lipids has recently been accepted as a common etiology of chronic liver diseases and a risk factor for metabolic syndrome [3,4,5,6,7,8].

Autophagy is a highly regulated, destructive process in which unnecessary or malfunctioning cellular components are ultimately degraded in lysosomes, enabling cells to recycle these materials [9]. The role of autophagy in lipid dysregulation and NAFLD pathogenesis is emerging. During the early stages of NAFLD, autophagy is activated due to an acute increase in lipid availability, attenuating lipid accumulation within the liver. However, long-term consumption of a high-fat diet impairs hepatic autophagy, leading to the accumulation of lipids [10]. The autophagy of lipid droplets, termed “lipophagy”, is a crucial mechanism of lipid mobilization, that regulates lipid homeostasis in hepatocytes [11]. Inhibiting autophagy in hepatocytes results in an increased size and number of lipid droplets [12], and is linked to the development of fatty liver and insulin resistance [9,13].

Red peppers (*Capsicum annuum* L.) have been used as traditional food pigments, spices, and medicines since ancient times [14,15,16,17]. Several studies suggest that capsaicin predominantly accounts for the beneficial effect of peppers [18,19,20,21,22]; however, capsaicin is predominantly present in the placenta of red pepper fruit [15]. Pepper seeds, the major waste products of pepper processing, rarely contain capsaicin [15]. Our preliminary results showed that red pepper seed (RPS) prevented adipocyte differentiation and lipid accumulation in 3T3-L1 cells via AMPK activation [23]. Additional studies from our group have also reported that RPS effectively attenuated HFD-induced body weight, hyperlipidemia, and insulin resistance by inhibiting adipogenic transcription factors [24]. However, the inhibitory effect of RPS on hepatic lipid accumulation, particularly autophagy activation, has not yet been demonstrated. In this study, we focus on hepatic lipid metabolism and autophagy activation using C57BL/6 mice [24] and a cell model of oleic acid (OA)-induced hepatic steatosis. Our results showed that RPS reduced lipogenesis-associated protein expressions and lipid accumulation via autophagy activation in the liver of HFD-fed animals and cell model of OA-induced steatosis. Furthermore, we demonstrated that reduced lipogenesis by activating autophagy was associated with AMPK activation.

## 2. Materials and Methods

### 2.1. Extract Preparation

The water extract of RPS was prepared in accordance with previous methods [23]. Briefly, 200 g of RPS powder was soaked in 1.8 L deionized water at 60 °C for 24 h. The extracts were then centrifuged, evaporated, and freeze-dried. We then quantitatively analyzed the amounts of icariside E_5_ and vanilloyl icariside E_5_ [25] and kept them at −70 °C until use.

### 2.2. Animals

Five-week-old male C57BL/6 mice were randomly divided into four groups (n = 8 per group): normal diet control (ND-C, AIN-93G diet composed of 15.8% kcal fat, Research Diets Inc., New Brunswick, NJ, USA), high-fat diet control (HFD-C, 60% kcal fat, Research Diets Inc., New Brunswick, NJ, USA), or HFD supplemented with RPS (100 or 200 mg/kg body weight). The mice were administered distilled water or RPS via oral gavage once daily for 13 weeks. The relative liver weight was calculated as the liver weight (g) divided by the final body weight (g). All animal experiments were approved by the Institutional Animal Care and Use Committee of Mokpo National University (MNU-IACUC-2016-006).

### 2.3. Determination of Serum AST and ALT levels

At the end of the experiments, blood was collected and centrifuged (2000× g, 20 min). Aspartate aminotransferase (AST) and alanine aminotransferase (ALT) levels were analyzed using an automated chemistry analyzer (Beckman Coulter Inc., Brea, CA, USA) according to the manufacturer’s instructions.

### 2.4. Measurement of Hepatic TG Level

Lipid extraction from the liver was determined a modified Folch method [26]. Briefly, 100 mg frozen liver tissues (n = 6) were homogenized with 0.9% NaCl solution. The liver homogenate was mixed with a chloroform: methanol (2:1) solution and incubated at room temperature for 30 min. After incubation, the samples were centrifugated, filtrated, and heated. Once completely drying, they were dissolved in chloroform/propanol and was determined using a TG measurement kit (AM147S-K, Asan-set, Seoul, Korea) according to the manufacturer’s instructions.

### 2.5. Cell Culture and Cell Viability Assay

Human hepatoma HepG2 cells obtained from the American Type Culture Collection (ATCC, Manassas, VA, USA) were maintained in Dulbecco’s Modified Eagle’s Medium (DMEM, Lonza, Basel, Switzerland) containing 10% fetal bovine serum (FBS, Gibco, Grand Island, NY, USA) and antibiotics at 37 °C in a humidified 5% CO_2_ atmosphere. To measure cell viability, HepG2 cells were plated in a 96-well plate at a density of 1 × 10⁴ cells/well. After 24 h, the spent medium was replaced with fresh medium containing 0, 100, or 200 μg/mL of RPS extract. After another 24 h of incubation, the cells were incubated with 3-(4,5-dimethylthiazol-2-yl)-2,5-diphenyltetrazolium bromide (MTT, Sigma-Aldrich, St. Louis, MO, USA) and the absorbance was measured. Cell viability was presented as a percentage of the control for each group.

### 2.6. Establishment of an Oleic Acid-Induced Hepatic Steatosis HepG2 Cell Model

The effects of RPS were investigated using an OA-induced hepatic steatosis cell model. Briefly, HepG2 cells were incubated in serum-free medium overnight and were replaced with a high-glucose (30 mM) medium in the absence or presence of 1 mM of OA (Sigma-Aldrich) for 24 h. After successfully producing the steatosis model, the cells were treated with the vehicle (PBS, Gibco) or RPS (100 or 200 μg/mL) for an additional 24 h with OA. To measure the autophagic flux, the OA-induced steatosis cells were treated with an autophagy inhibitor, 3-methyladenine (3-MA, 1 mM, Sigma-Aldrich) for 1 h. The cells were then incubated with OA and RPS for 24 h additionally.

### 2.7. Cell Transfection with siRNA

HepG2 cells were transfected with non-targeting small interfering RNA (siRNA) and siRNA targeting AMPKα1/α2 (Santa Cruz Biotechnology, Dallas, TX, USA) using Lipofectamine 3000 (Invitrogen, Carlsbad, CA, USA) according to the manufacturer’s instructions. After 12 h of transfection, the cells were transferred back to the culture medium for subsequent experiments.

### 2.8. Western Blot Analysis

Liver tissues and cell lysates were prepared in a radioimmune precipitation assay (RIPA) buffer (Thermo Fisher Scientific, Waltham, MA, USA) with a 1% protease inhibitors cocktail (Thermo Fisher Scientific) and phosphatase inhibitor (Thermo Fisher Scientific). Protein concentrations were determined using the Bradford method (Sigma-Aldrich) and equal amounts of protein (40 μg) were separated on 10% SDS-PAGE gels and transferred onto a nitrocellulose membrane (Millipore, Billerica, MA, USA). The membranes were blocked and incubated with primary antibodies overnight at 4 °C. All antibodies used are summarized in Table S1. The membranes were washed with Tris-buffered saline containing Tween 20 (TBS-T) and incubated with the corresponding horseradish peroxidase-conjugated secondary antibodies [goat anti-rabbit or goat anti-mouse (1:2000)]. Blots were then developed using a chemiluminescent substrate (IMGENEX, San Diego, CA, USA) and detected using a UVP imaging system (UVP, Upland, CA, USA). Protein bands were quantified using AlphaEaseFC 4.0 (Alpha Innotech, San Leandro, CA, USA).

### 2.9. Oil Red O (ORO) Staining

For the cell study, HepG2 cells were fixed with 10% paraformaldehyde for 30 min and then stained with ORO (Sigma-Aldrich) for 1 h. ORO-stained cells were observed under a microscope (magnification 10×). Thereafter, lipids were extracted using isopropanol and transferred to a clear 96-well plate. Absorbance was measured at 540 nm using a microplate reader (Multiskan EX, Thermo Fisher Scientific, Waltham, MA, USA).

To assess fat deposition in the mouse liver, the left lobe of liver tissue was embedded in the optimal cutting temperature (OCT) compound (Scigen, CA, USA) and 10 μm thick sections were created using a cryostat (Leica Biosystems, Wetzlar, Germany). Cryostat sections were stained with ORO (in 70% isopropyl alcohol) and observed under a microscope (magnification 20×).

### 2.10. Statistical Analysis

Statistical analyses were performed using the SPSS 23.0 software. Data were analyzes using a one-way analysis of variance (ANOVA), followed by Duncan’s multiple comparison test. Statistical significance was set p-value less than 0.05. Data are presented as the mean ± SEM.

## 3. Results

### 3.1. RPS Decreased Hepatic Toxicity, Lipid Droplet Accumulation, and Lipogenic-Associated Protein Expression in HFD-Fed Mice

**Figure 1:**
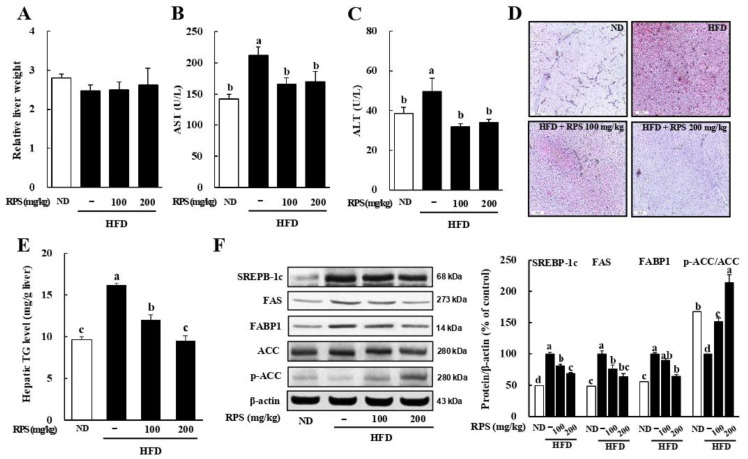
Red pepper seed (RPS) decreased lipid droplets in the liver of HFD-fed mice. C57BL/6 mice (n = 8) were fed a normal or a high-fat diet and were treated with RPS by oral gavage with RPS in distilled water at the concentration of either 0, 100, or 200 mg/kg body weight for 13 weeks. (**A**) Relative liver weight. (**B**) Serum AST levels. (**C**) Serum ALT levels. (**D**) Representative ORO-stained frozen liver section. (**E**) Hepatic TG level. (**F**) Expression of lipogenesis-related proteins. β-actin was used as a control for quantification. Different letters are significantly different by Duncan’s multiple range test (*p* < 0.05). ND, normal diet with oral administration of PBS; HFD, high-fat diet with oral administration of PBS; HFD + RPS100, high-fat diet with oral administration of RPS 100 mg/kg body weight; HFD + RPS200, high-fat diet with oral administration of RPS 200 mg/kg body weight.

Our previous results showed that RPS treatment effectively decreased the final body weight of mice fed with a HFD [24]. The relative liver weight was not different among the groups, which was the same for liver weight as well (Figure 1A and Table S2). Serum AST and ALT levels, which are indicators of liver toxicity, were significantly lower in the RPS group than in HFD-fed mice (Figure 1B,C). Next, we evaluated hepatic steatosis via ORO staining. The ORO-stained lipid droplets were dramatically higher in the HFD-C group than those in the ND-C group and was reduced by RPS treatment in HFD-fed mice (Figure 1D). Importantly, hepatic TG levels dramatically decreased in the RPS group compared to those in the HFD-C group (Figure 1E). Western blot analyses revealed that the RPS-induced suppression of lipid accumulation was accompanied by the downregulation of sterol regulatory element-binding protein 1c (SREBP-1c), fatty acid synthetase (FAS), and fatty acid-binding protein 1 (FABP1) expression, whereas upregulating acetyl-CoA carboxylase (ACC) phosphorylation in the liver (Figure 1F).

### 3.2. RPS Treatment Induced Autophagic Flux and AMPK Activation in the Livers of HFD-Fed Mice

**Figure 2:**
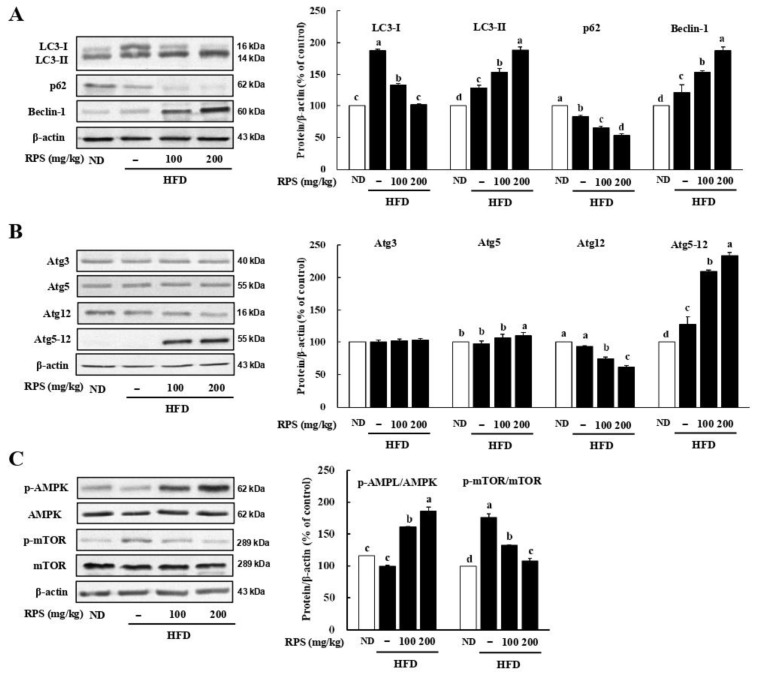
Red pepper seed elevated autophagy in the liver of HFD-fed mice. (**A**) Protein expression of LC3, p62, and Beclin-1. (**B**) Protein expression of Atg3, Atg5, Atg12, and Atg5-12. (**C**) Protein expression of AMPK/mTOR pathway. β-actin was used as a control for quantification. All data represent three independent biological replicates. Different letters are significantly different according to Duncan’s multiple range test (*p* < 0.05). ND, normal diet with oral administration of PBS; HFD, high-fat diet with oral administration of PBS; HFD + RPS100, high-fat diet with oral administration of RPS 100 mg/kg body weight; HFD + RPS200, high-fat diet with oral administration of RPS 200 mg/kg body weight.

To determine whether the inhibition of hepatic lipid accumulation corresponded with the increased autophagic flux, we measured the lysosomal turnover of the autophagosome marker microtubule-associated protein light chain 3 (LC3) in liver samples using immunoblotting. The expression level of LC3-Ⅱ in the liver was visibly increased by the treatment with RPS (Figure 2A). We also noted a decrease in p62 expression in the RPS-treated mice in comparison to that of HFD-C (Figure 2A). In addition, RPS-induced autophagic flux increases in the expression of pro-autophagy proteins including Beclin-1 (Figure 2A) and the autophagy-related 5–12 (Atg5-Atg12) conjugate (Figure 2B).

AMPK activation induces autophagy to attenuate hepatic steatosis by dampening de novo lipogenesis [27]. We assessed whether AMPK activation was induced by RPS, which inhibited lipogenesis and accelerated autophagy activation in HFD-fed animals. The p-AMPK/AMPK ratio was increased, whereas the p-mTOR/mTOR ratio was reduced compared to HFD-C mice (Figure 2C).

### 3.3. RPS Down-Regulated the Expression of Lipogenesis-Related Proteins in a Cell Model of OA-Induced Steatosis

**Figure 3:**
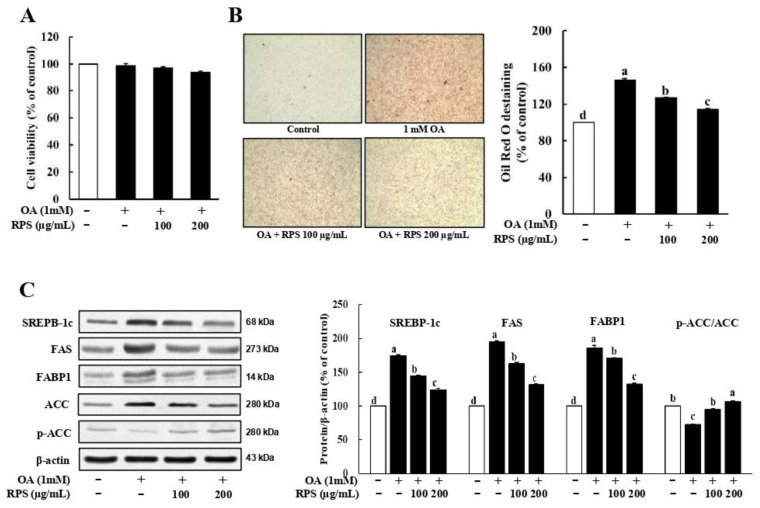
Red pepper seed suppressed lipogenesis in a cell model of OA-induced steatosis. HepG2 cells were incubated with OA for 24 h, and then treated with RPS (0, 100, or 200 μg/mL) for additional 24 h. (**A**) Cell viability. (**B**) Oil Red O staining. (**C**) Expression of lipogenesis-related proteins. β-actin was used as a control for quantification. All data represent three independent biological replicates. Different letters are significantly different according to Duncan’s multiple range test (*p* < 0.05).

To investigate the inhibitory effect of RPS in an OA-induced hepatic steatosis cell model, HepG2 cells were pre-treated with OA for 24 h and then treated with RPS for another 24 h. The concentrations of OA and RPS showed no apparent toxicity to HepG2 cells (Figure 3A). Next, we stained lipid droplets with ORO and quantified the lipid droplets by measuring the absorbance of OA-induced steatosis in HepG2 cells. Exposure to high levels of glucose and fatty acid, particularly in the OA-treated group, visibly increased lipid accumulation in HepG2 cells. However, the RPS treatment dramatically attenuated triglyceride content compared to OA-treated control cells (Figure 3B). Consistent with the reduced lipid accumulation, the OA treatment significantly increased the expression of lipogenesis-related proteins, including SREBP-1c, FAS, and ACC, compared with the control, which was dose-dependently reversed by the RPS treatment. (Figure 3C).

### 3.4. RPS Up-Regulated Autophagic Flux in a Cell Model of OA-Induced Steatosis

**Figure 4:**
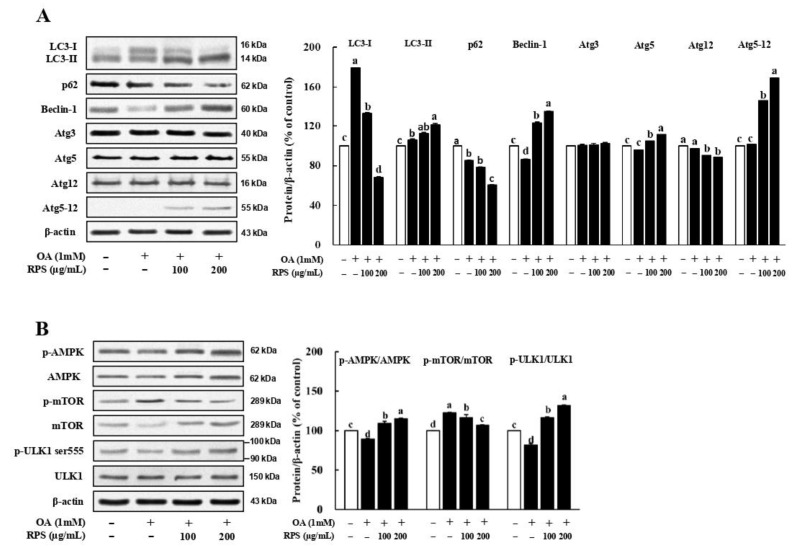
Red pepper seed increased autophagy flux in a cell model of OA-induced steatosis. HepG2 cells were incubated with OA for 24 h, and then treated with RPS (0, 100, or 200 μg/mL) for additional 24 h. (**A**) Protein expression of autophagy. (**B**) Protein expression of AMPK/mTOR/ULK pathway. β-actin was used as a control for quantification. All data represent three independent biological replicates. Different letters are significantly different according to Duncan’s multiple range test (*p* < 0.05).

The expression of LC3-II, a standard autophagosome marker, was obviously upon treatment with high concentrations of the RPS in OA-induced steatosis cells. In addition, the expression of p62, another key protein of autophagy, was significantly reduced after RPS treatment (Figure 4A). Furthermore, we found that RPS-induced autophagy in OA-treated HepG2 cells was associated with the increased expression of pro-autophagic proteins, such as Atg5, Atg12-Atg5 conjugate, and Beclin-1. (Figure 4A). Notably, the expression of the Agt12-Agt5 conjugate, which plays a critical role in LC3 lipidation and autophagosome formation, was substantially increase upon RPS treatment [28,29]. A significant decrease in the p-AMPK/AMPK ratio was also observed in OA-treated HepG2 cells, which was attenuated by RPS treatment. The p-mTOR/mTOR ratio was downregulated by the RPS treatment in a dose-dependent manner in OA-treated HepG2 cells (Figure 4B). The phosphorylation of UNC-5-like autophagy activating kinase 1 (ULK1) at Ser555 is an important downstream effector of AMPK activation. The results showed that the p-ULK1 (Ser555)/ULK1 ratio increased in a dose-dependent manner following RPS treatment (Figure 4B).

### 3.5. Inhibition of Autophagy Abolished the RPS-Induced Suppression of Lipogenesis in a Cell Model of OA-Induced Steatosis

**Figure 5:**
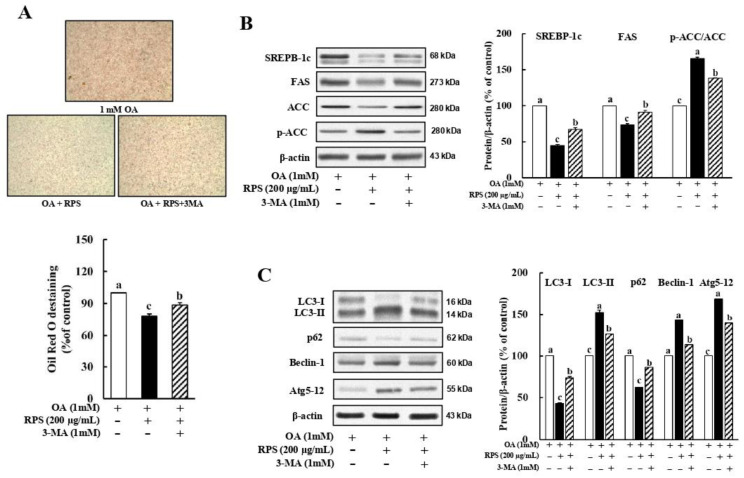
Red pepper seed inhibited steatosis through reinforcing autophagy in a cell model of OA-induced steatosis. HepG2 cells were incubated with OA for 24 h, and then treated with autophagy inhibitor, 3-MA, and RPS (0, or 200 μg/mL) for additional 24 h. (**A**) Oil Red O staining. (**B**) Protein expression pattern of lipogenesis. (**C**) Protein expression of autophagy. β-actin was used as a control for quantification. All data represent three independent biological replicates. Different letters are significantly different according to Duncan’s multiple range test (*p* < 0.05).

To examine whether autophagy plays a role in the RPS-induced suppression of lipogenesis, OA-treated HepG2 cells were incubated with 200 μg/mL RPS in the presence or absence of the autophagy inhibitor, 3-MA (1 mM). As expected, the 3-MA-induced inhibition of autophagy dramatically reversed the RPS-induced inhibition of lipid accumulation (Figure 5A), expression of SREBP1-c and FAS, and the ACC (Figure 5B). In addition, combined treatment with 3-MA and RPS abolished the RPS-induced autophagic flux, as assessed via the LC3-II and p62 expression, as well as the expression of Atg5-Atg12 conjugate and Beclin-1 (Figure 5C). Collectively, these results indicate that RPS treatment could help prevent hepatic lipid accumulation by activating autophagy.

### 3.6. RPS-Induced Autophagy Activation Is Associated with the AMPK/mTOR Signaling in a Cell Model of OA-Induced Steatosis

**Figure 6:**
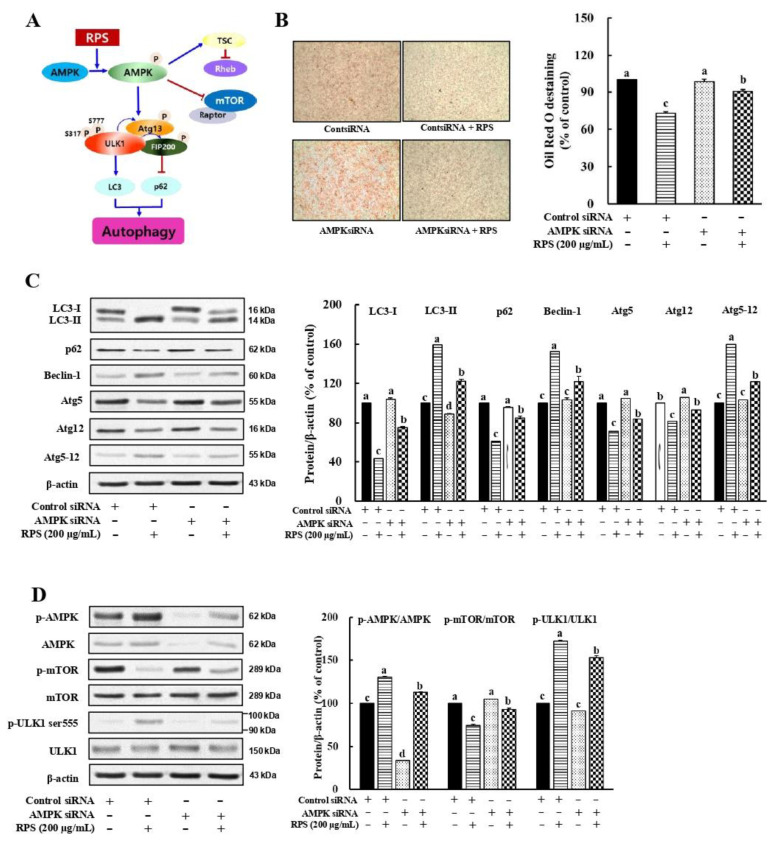
Red pepper seed-induced autophagy is AMPK dependent in a cell model of OA-induced steatosis. HepG2 cells were incubated with either control or AMPK siRNA for 24 h, and then treated with RPS (0 or 200 μg/mL) for additional 24 h. (**A**) Experimental hypothesis. (**B**) Oil Red O staining. (**C**) Protein expression of autophagy. (**D**) Expression of proteins associated with AMPK/mTOR/ULK pathway. β-actin was used as a control for quantification. All data represent three independent biological replicates. Different letters are significantly different according to Duncan’s multiple range test (*p* < 0.05).

A dose-dependent increase in the AMPK phosphorylation was observed after RPS treatment in OA-treated HepG2 cells (Figure 4B). mTOR signaling, which is known to suppress autophagosome formation [30], was inhibited by the RPS treatment (Figure 4B). These results led us to speculate whether RPS-induced autophagy was associated with AMPKα1 activation (Figure 6A). Next, we used specific AMPKα1 siRNA for the temporary knockdown AMPKα1 expression in an OA-induced hepatic steatosis cell models. The alleviating effect of RPS on lipid droplet deposition in OA-induced steatosis cells was significantly weakened by the knockdown of AMPKα1 expression (Figure 6B). In addition, upon AMPK knockdown, RPS treatment drastically decreased the LC3-II expression compared to cells treated with control siRNA (Figure 6C). Following the LC3-II expression results, the protein expression of p62 showed a significant increase following RPS treatment upon AMPK knockdown (Figure 6C). Moreover, expressions of Beclin-1 and Atg5-12 were effectively inhibited by RPS treatment upon AMPK knockdown compared to RPS treatment alone, indicating that the AMPK siRNA antagonized the RPS-induced autophagic flux as well as the RPS-induced suppression of lipid accumulation (Figure 6C). Furthermore, we found that RPS treatment in the presence of siAMPK increased the ratio of p-mTOR/mTOR and decreased the ratio of p-ULK1/ULK1 compared to that in the presence of control siRNA (Figure 6D). This result indicates that RPS induces autophagy and attenuates liver lipid accumulation by activating AMPK signaling.

## 4. Discussion

Capsaicin and capsicoside G have been extensively studied in metabolic diseases including obesity, insulin resistance, and fatty liver disease [20,21,31]. Their health benefits were thought to be conferred through an anti-inflammatory effect, rather than autophagic activation. In addition, our previous study revealed a direct and profound inhibitory effect of RPS on obesity and insulin resistance in HFD-fed mice [24]. However, the effects and potential mechanisms of RPS on hepatic lipid accumulation and the involvement of autophagy are not yet completely understood. Here, we demonstrated that RPS inhibited hepatic lipid accumulation through the autophagy-mediated downregulation of lipogenesis. Furthermore, we provide the evidence that RPS protects against hepatic lipid accumulation through autophagy activation, which is dependent on AMPK/mTOR signaling in vitro and in vivo.

In 1998, Day and James proposed the “two-hit” theory to describe the development of NAFLD [32]. According to this hypothesis, fat accumulation in the liver is the “first hit. Excessive fat accumulation leads to oxidative stress, proinflammatory cytokines, and insulin resistance, which is the “second hit”. Thus, controlling lipid accumulation in the liver is critical for preventing NAFLD. In this study, we showed that RPS effectively reduced hepatic lipid accumulation in a HFD-induced obesity animal model. We also confirmed that RPS reversed the OA-induced lipid accumulation in HepG2 cells. Moreover, RPS treatment increased the expression ratio of p-AMPK/AMPK, thereby reducing the expression of ACC, in the lipogenic gene in vivo (Figure 1) and in vitro (Figure 3). Multiple studies have suggested that AMPK phosphorylation suppresses triglyceride synthesis through the rapid phosphorylation of ACC [33,34,35]. Phosphorylated ACC decreases the carboxylation of acetyl-CoA to malonyl-CoA and activates carnitine palmitoyl-transferase 1 (CPT-1), thereby reducing de novo lipogenesis [35] and increasing the oxidation of long-chain fatty acids [36]. Additionally, RPS inhibited SREBP1-c and FAS expressions, as well as mTOR phosphorylation. The activation of mTORC1 in the liver, which is inhibited by AMPK, increases the expression of SREBP-1c, a well-known transcription factor for FAS [37]. Collectively, these results indicate that RPS reduced de novo lipogenesis via the AMPK/mTOR pathway.

Growing evidence suggests that defective autophagy in hepatocytes is a possible pathophysiological mechanism for the development of NAFLD [38,39]. Therefore, the activation of autophagy may be a promising strategy to attenuate hepatic lipid accumulation. The involvement of the AMPK/mTOR pathway in the RPS-induced regulation of lipogenic proteins prompted us to explore whether autophagy activation is another mechanism for inhibiting lipid accumulation, because AMPK activation is necessary for activating autophagy and inhibiting lipid deposition [37,40,41]. We also demonstrated that RPS increased autophagic flux and pro-autophagic protein expression in OA-treated HepG2 cells (Figure 4) and in the livers of HFD-fed animals (Figure 2). Interestingly, our results using 3-MA, an inhibitor of autophagy activation, showed that autophagy activation is involved in the lipogenic regulation activity of RPS by inhibiting the expression of lipogenesis-related proteins (Figure 5).

Recently, Mei et al. [42] reported that isosteviol sodium, a sweetener isolated from Stevia rebaudiana, inhibited lipid deposition by enhancing autophagy via Sirt1/AMPK signaling. In addition, accumulating evidence suggests that the AMPK/mTOR pathway plays a central role in the regulation of autophagy initiation in NAFLD [43,44,45,46,47]. These findings indicate that enhancing autophagy via AMPK/mTOR signaling may play a pivotal role in attenuating the development of NAFLD. However, phosphorylated AMPK can directly activate Beclin-1 or by inhibiting ULK1. Beclin-1 is located at the site of autophagosome initiation and contributes to the activation of autophagy components such as the Atg12-Atg5 conjugate, a key component of autophagy that interacts with Atg16 and functions upstream of LC3 lipidation and its autophagosome target [48,49,50]. Here, we found that RPS increased autophagy via AMPK activation, which was abolished by AMPK knockdown (Figure 6).

In conclusion, the present study showed that RPS inhibits hepatic lipid accumulation via AMPK-mediated autophagy. Thus, our findings suggest that RPS may have an inhibitory effect on ectopic lipid deposition both in vitro and in vivo, offering a potentially promising new approach for the treatment of hepatic lipid accumulation.

## Supplementary Materials

The following supporting information can be downloaded at: https://www.mdpi.com/article/10.3390/nu14204247/s1, Table S1: List of primary antibodies, Table S2: Liver weight in HFD-fed with RPS treatment.
