# Supplementary material for: Red Pepper Seeds Inhibit Hepatic Lipid Accumulation by Inducing Autophagy via AMPK Activation
Source: Nutrients. 2022 Oct 12;14(20):4247. doi: 10.3390/nu14204247 (PMC9608681; doi:10.3390/nu14204247)
Supplement: Supplementary file 1 [file nutrients-14-04247-s001.zip › nutrients-1903666-supplementary.pdf]

nutrients MDPI

Supplementary Materials

Red Pepper Seeds Inhibit Hepatic Lipid Accumulation by Inducing Autophagy via AMPK Activation

Young-Hyun Lee [1,†], Hwa-Jin Kim [1,†], Mikyoung You [2,†] and Hyeon-A Kim [1,*]

[1] Department of Food and Nutrition, Mokpo National University, Muan-gun 58554, Korea
[2] Nutrition Research Institute, University of North Carolina, Chapel Hill, NC 28081, USA
[†] These authors contributed equally to this work.

Inventory of Supplemental Information

Supplemental Tables

- **Table S1.** List of primary antibodies
- **Table S2.** Liver weight in HFD-fed with RPS treatment.

Table S1. List of primary antibodies.

Antibody	Host	Dilution	Company	Catalog no.
SREBP-1c	Rabbit	1:1000	Santa Cruz Biotechnology	SC-366
FAS	Rabbit	1:1000	Cell Signaling	3180
FABP1	Rabbit	1:1000	Cell Signaling	3544
p-ACC	Rabbit	1:1000	Cell Signaling	3661
ACC	Rabbit	1:1000	Cell Signaling	3662
LC3	Rabbit	1:1000	Cell Signaling	2775
p62	Rabbit	1:1000	Cell Signaling	23214
Beclin 1	Rabbit	1:1000	Cell Signaling	3495
Atg3	Rabbit	1:1000	Cell Signaling	3415
Atg5-12	Rabbit	1:1000	Cell Signaling	4180
p-AMPK	Rabbit	1:1000	Cell Signaling	2535
AMPK	Rabbit	1:1000	Cell Signaling	2532
p-mTOR	Rabbit	1:1000	Cell Signaling	5536
mTOR	Rabbit	1:1000	Cell Signaling	2983
Atg5	Rabbit	1:1000	Cell Signaling	12994
Atg12	Rabbit	1:1000	Cell Signaling	4180
β-actin	Mouse	1:1000	Santa Cruz Biotechnology	sc-47778

Table S2. Liver weight in HFD-fed with RPS treatment.

Groups	Liver weight (g)
ND-C	0.92 ± 0.03
HFD-C	1.00 ± 0.03
HFD_RPS100	0.96 ± 0.02
HFD_RPS200	0.92 ± 0.05
